# Cervical cancer survival times in Africa

**DOI:** 10.3389/fpubh.2022.981383

**Published:** 2022-11-09

**Authors:** Emmanuel Kwateng Drokow, Fangnon Firmin Fangninou, Clement Yaw Effah, Clement Agboyibor, Yunfeng Zhang, Francisca Arboh, Marie-Anne Deku, Wu Xinyin, Yue Wang, Kai Sun

**Affiliations:** ^1^Department of Radiation Oncology, Henan Provincial People's Hospital, People's Hospital of Zhengzhou University, Zhengzhou, China; ^2^State Key Laboratory of Pollution Control and Resource Reuse, Key Laboratory of Yangtze River Water, Ministry of Education, College of Environmental Science and Engineering, Tongji University, Shanghai, China; ^3^College of Public Health, Zhengzhou University, Zhengzhou, China; ^4^School of Pharmaceutical Sciences, Zhengzhou University, Zhengzhou, China; ^5^Department of Obstetrics and Gynaecology, Henan Provincial People's Hospital, People's Hospital of Zhengzhou University, Zhengzhou, China; ^6^School of Management Science and Engineering, Jiangsu University, Zhenjiang, China; ^7^Binzhou Medical University, Yantai, China; ^8^Xiangya School of Public Health, Central South University, Changsha, China; ^9^Department of Hematology, Henan Provincial People's Hospital, People's Hospital of Zhengzhou University, Zhengzhou, China

**Keywords:** cervical cancer, survival rate, Africa, meta-analysis, systematic review

## Abstract

**Objective:**

Accessibility to quality healthcare, histopathology of tumor, tumor stage and geographical location influence survival rates. Comprehending the bases of these differences in cervical cancer survival rate, as well as the variables linked to poor prognosis, is critical to improving survival. We aimed to perform the first thorough meta-analysis and systematic review of cervical cancer survival times in Africa based on race, histopathology, geographical location and age.

**Methods and materials:**

Major electronic databases were searched for articles published about cervical cancer survival rate in Africa. The eligible studies involved studies which reported 1-year, 3-year or 5-year overall survival (OS), disease-free survival (DFS) and/or locoregional recurrence (LRR) rate of cervical cancer patients living in Africa. Two reviewers independently chose the studies and evaluated the quality of the selected publications, in accordance with the Preferred Reporting Items for Systematic Reviews and Meta-analyses (PRISMA-P). We used random effects analysis to pooled the survival rate across studies and heterogeneity was explored *via* sub-group and meta-regression analyses. A leave-one-out sensitivity analysis was undertaken, as well as the reporting bias assessment. Our findings were reported in accordance with the Preferred Reporting Items for Systematic Reviews and Meta-analyses (PRISMA-P).

**Results:**

A total of 16,122 women with cervical cancer were covered in the 45 articles (59 studies), with research sample sizes ranging from 22 to 1,059 (median = 187.5). The five-year overall survival (OS) rate was 40.9% (95% CI: 35.5–46.5%). The five-year OS rate ranged from 3.9% (95% CI: 1.9–8.0%) in Malawi to as high as 76.1% (95% CI: 66.3–83.7%) in Ghana. The five-year disease-free survival rate was 66.2% (95% CI: 44.2–82.8%) while the five-year locoregional rate survival was 57.0% (95% CI: 41.4–88.7%).

**Conclusion:**

To enhance cervical cancer survival, geographical and racial group health promotion measures, as well as prospective genetic investigations, are critically required.

## Introduction

Cervical cancer is one of the most common female cancer affecting women across the globe. In 2020, 19.3 million cases of cancer were newly diagnosed worldwide (1, 2). Cervical cancer accounted for 3.1% (604,127) of the newly diagnosed cancer with a mortality of 3.4% (341,831) (2). Cervical cancer is a sexually transmitted disease caused by the Human Papilloma Virus (HPV) Types 18 and 16 (2). While cervical cancer prevalence is lowest in high-income nations (HINs), the same cannot be said in low and middle-income countries (LMICs) due to the high prevalence rate. The number of disability adjusted life (DALYs) caused by cervical cancer increased by “44.99% to 8,955.01 × 10^3^ (95% UI: 7,547.73 × 103 to 9,978.46 × 10^3^)” between 1990 and 2019 (3). Cervical cancer mortality rates are significantly lower in developed countries than in underdeveloped and developing countries. For instance, in 2020, mortality rates of cervical cancer in terms of age-standardization in developed countries was 1.6 per 100,000 females in comparison to 28.6 per 100,000 females in underdeveloped countries (2). Again, <30% LMICs have a nationwide HPV vaccination program while over 80% HICs have rolled out effective nationwide HPV vaccination program (2, 4, 5). Poverty and Human Development Index (HDI) have shown to contribute to these disparities in cervical cancer mortality and incidence (2, 6). Khazaei et al. reported that Cervical cancer mortality and incidence were inversely correlated with both gender disparity and low levels of human development (5). The incidence of cervical cancer is reduced by 20% and the mortality risk related to this cancer is reduced by 33% with each unit rise in HDI of 0.2 (6). Poverty and low HDI have resulted in several cervical cancer patients being diagnosed at an advanced stage of the diseases and others seeking for herbal treatment and spiritual intervention.

Of the 604,127 newly diagnosed cases of cervical cancer in 2020, about −80% occurred in Africa with Malawi having the highest diagnosed number of cases (2). Cervical cancer prevalence and death rates are rising throughout Africa. Eastern, Southern, Middle and Western Africa are among the top five regions with the highest incidence and mortality of cervical cancer cases (2). Accessibility to quality healthcare, histopathology of tumor, tumor stage and geographical location influence survival rates. Comprehending the basis of these differences in cervical cancer survival rate, as well as the variables linked to poor prognosis, is critical to improving survival. Cervical cancer overall survival rates for the whole African continent have not been comprehensively explored to the best of our knowledge. We analyzed the available studies on cervical cancer survival rates for the entire African continent in this study. The primary goal of this review was to determine historical and current cervical cancer patient survival rates across the African countries, as well as to identify any potential sources of variability between studies. These findings could aid in the development of future therapies aimed at prolonging survival in African women with cervical cancer.

## Methods and materials

### Registration of study protocol

The guidelines for Preferred Reporting Items for Systematic Reviews and Meta-analyses (PRISMA-P) (Supplementary Table S1) (7) was followed and the study protocol was registered with PROSPERO with registration ID, CRD42022316197.

### Study location

Africa, the world's second-largest continent (after Asia), accounts for around one-fifth of the planet's total land area (8). The Atlantic Ocean borders the continent on the west, the Mediterranean Sea on the north, the Indian Ocean and the Red Sea on the east, and the mingling waters of the Indian and Atlantic oceans on the south (9). Africa's overall land area is roughly 11,724,000 square miles (30,365,000 square km), with a north-south distance of 5,000 miles (8,000 km) and an east-west distance of 4,600 miles (7,400 km. Africa is divided into five primary geographical sub-regions or zones (10) (Table 1). The world's second-largest continent is home to 1.37 billion people, approximately 14% of the worldwide population in 2021 (11).

**Table 1:** The five sub-regions in Africa.

**Sub-regions in Africa**	**Names of countries**
Northern Africa (7)	Algeria, Egypt, Libya, Morocco, Sudan, Tunisia, and Western Sahara
Central or Middle African countries (9)	Angola, Cameroon, Central African Republic, Chad, Congo Republic–Brazzaville, Democratic Republic of Congo, Equatorial Guinea, Gabon, and São Tomé & Principe
Southern Africa countries (5)	Botswana, Lesotho, Namibia, South Africa, and Swaziland
East African countries (19)	Burundi, Comoros, Djibouti, Ethiopia, Eritrea, Kenya, Madagascar, Malawi, Mauritius, Mozambique, Réunion, Rwanda, Seychelles, Somalia, Somaliland, Tanzania, Uganda, Zambia, and Zimbabwe
Western Africa (17)	Benin, Burkina Faso, Cape Verde, Côte D'Ivoire, Gambia, Ghana, Guinea, Guinea-Bissau, Liberia, Mali, Mauritania, Niger, Nigeria, Senegal, Sierra Leone, and Togo

As of July 2022, there were 425 teletherapy equipment being used by 257 radiotherapy centers in Africa, according to the IAEA's Directory of Radiotherapy Centers (DIRAC). One hundred and five brachytherapy equipment's are currently being used in Africa for cervical cancer treatment (12).

### Eligibility criteria

The inclusion criteria included:

- Published articles related to cervical cancer survival rate from any African country.
- Articles which reported at least 1-year (OS), 3-year (OS) rate or 5-year OS AND/ OR Locoregional recurrence (LRR), disease free survival (DFS) of cervical cancer patients living in Africa.
- Conference proceedings, abstracts, original and published articles which report least 1-year (OS), 3-year (OS) rate or 5-year OS AND/ OR Locoregional recurrence (LRR), disease free survival (DFS) of cervical cancer patients living in Africa.

Regarding regional/multi-countries studies, articles were considered eligible when the extraction of data on 1-, 3-, or 5-year OS AND/OR LRR and DFS relating to African nations is accessible. In circumstances where there are many publications about the same study, the one with the most comprehensive and relevant material was selected.

### Exclusion criteria

The exclusion criteria included:

1. Published article related to other gynecological cancers.
2. Articles which report <1-year over-survival (OS) AND/OR LRR and DFS.
3. Unpublished articles, preprints manuscripts, commentaries, review articles and expert opinion.

### Searching strategy

Eligible articles were accessed *via* prominent electronic databases (EMBASE, Web of Science, Medline, PubMed and Ovid) along with African Journals Online. Google Scholar was additionally used to search for gray literature. The PICO strategy, which includes “P” for population, “I” for exposure or intervention, “C” for comparator, and “O” for outcomes, drive the search terms (13). The keywords “cervical cancer patient”, “women”, “females” was used for the population. The keywords “concurrent chemoradiotherapy”, “radiation therapy”, “Brachytherapy”, “Surgery” was used for the intervention. There were no “comparator” keywords. The terms “survival”, “OS”, “DFS”, “LRR”, “over survival rate”, “disease-free survival”, “Locoregional recurrence”, “1-year”,” 3-year” and “5-year OS” was used for outcome. The relevant keywords for the “context” comprised “Africa” and the names of various African nations. The results of the various searching words were merged using the relevant Boolean operators “AND” and “OR” (Supplementary Table S1). Furthermore, hand searching of the references of the selected studies was conducted to further identify eligible studies.

### Study selection

Two reviewers separately handled the article selection utilizing the Covidence application (14), which was built specifically to eliminate all duplicated publications retrieved from various databases. The abstracts and titles of publications found through the search approach were reviewed to exclude publications that were ineligible (7). All full-text articles of possibly eligible articles were retrieved and assessed extensively to see if they fit the criteria for inclusion. Any disagreements amongst reviewers were flagged by the systematic review management software, which was handled by mutual agreement.

### Extraction of data

The data extracted included median or mean age at diagnosis, year of diagnosis, study population, study design, publication date, origin of study, and sample size. If a study's racial composition was not reported, the population was presumed to be the same as that of the location where the article was published from. North African nations was assumed to have a primarily non-black population.

The 1-, 3-, and 5-year OS, LRR and DFS values for all eligible study was extracted. If the rate of survival was not stated, we used Tierney and colleagues' approach to estimate them from the original Kaplan Meier curves (15). DEK and FF cross-validated the data when it was entered independently.

### Quality assessment

Two reviewers independently assessed the quality of study by adopting and modifying the Newcastle Ottawa Scale (NOS) which has been validated for retrospective and prospective studies (16). The reviewers assessed the quality of study data from three domains: Selection, Comparability and Outcome. Each article was assigned a quality score between 0 to 9 (poor to good quality) depending on the total of those domains. Mutual agreement was used to addressed discrepancies in reviewers' judgments.

### Data analysis

After stabilizing the variability of each selected study utilizing the DerSimonian-Laird (DL) estimator, we pooled the 1-, 3-, and 5-year OS, DFS and LRR across the selected articles using a random effects model (17). This will reduced the effect of studies with extreme smaller or larger estimates on the pooled survival rates estimate. The Cochran's Q chi-squared test statistic (18) and the Thompson's and Higgins I^2^ statistic (1) was used to measure study heterogeneity. High, moderate, low and no levels of heterogeneity was represented by the cut-off I^2^ values of 75, 50, 25, and 0% respectively. Forest plots were used in representing the 95% confidence interval survival rates estimates of each selected study in addition to that of the pooled survival rate estimates.

To investigate the probable causes of heterogeneity, we conducted meta-regression and sub-group analyses. The sub-group analysis comprised of publication year, non-black African vs. black African (central, southern, western and eastern Africa), publication year, and age group. The meta-regression analysis necessitates the availability of at least five articles for each predictor in the model (19).

We also conducted sensitivity analysis to examined the robustness of our results, in which we investigated the effects of deleting one study at a time on the pooled estimate (20). If there were more than five studies involved, we used the Egger's test and funnel plot asymmetry to see if there was any reporting bias (21). In meta-analysis of survival rate studies, funnel plot was commonly employed to reveal publication bias visually (22). A plot of the effect sizes against their precisions or standard errors was frequently included in the graph (the inverse of standard errors). It may, however, be inappropriate for evaluating publication bias in meta-analysis of proportional studies with low percentage outcomes, where plot asymmetries may be incorrectly ascribed (23). For such cases, funnel plots showing log odds against study size or statistical analyses like the Egger's test was used to make a decision. Except for the leave-one-out influence analysis and other statistical analyses were done with Comprehensive Meta-Analysis (CMA) (24–26), with a 5% significance level.

## Results

### Study selection

**Figure 1:**
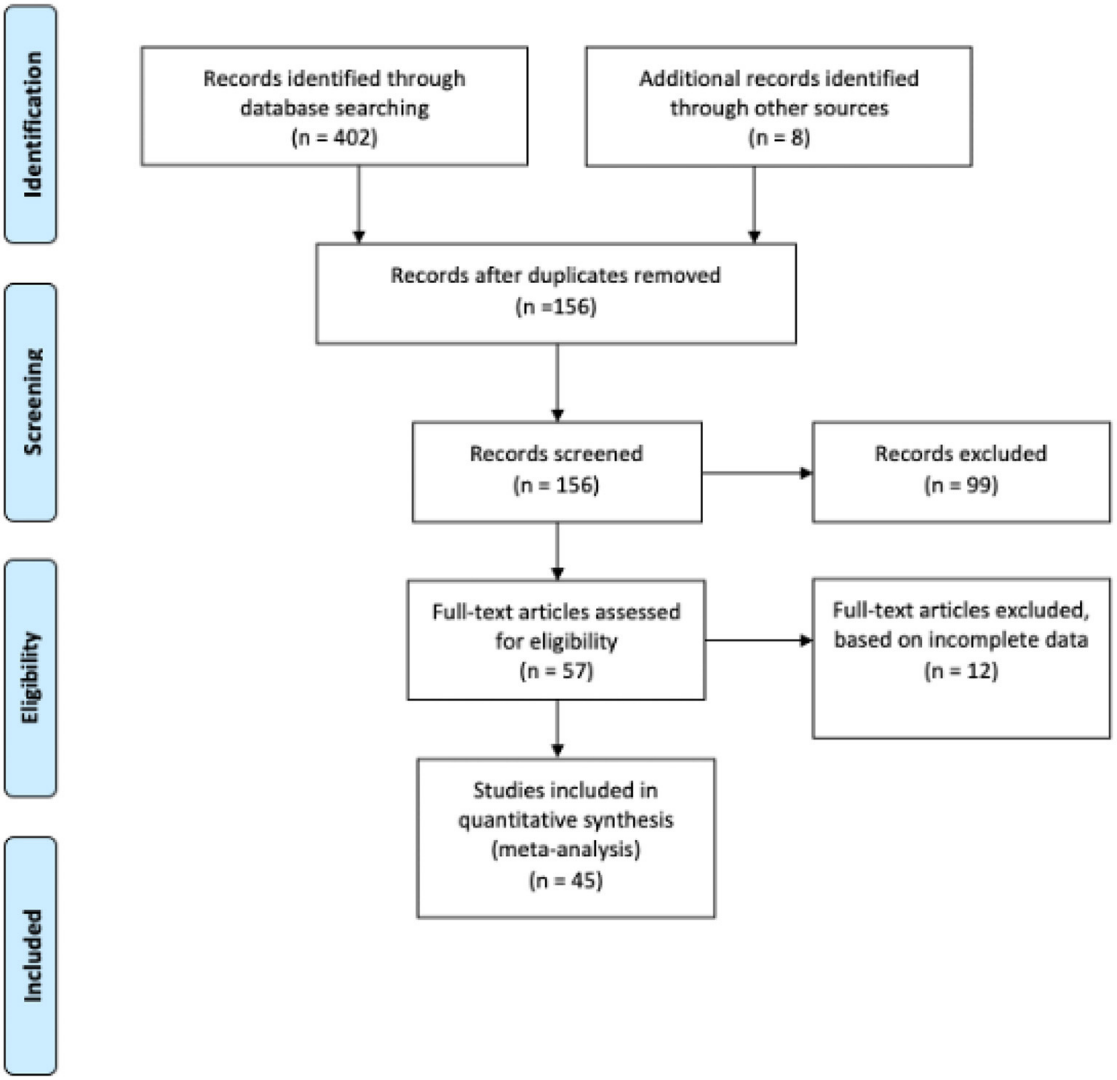
Flowchart of the selected studies.

Figure 1 shows the flowchart procedure of how articles were identified, screened, and included in our study. An extensive search for published studies from EMBASE, Web of Science, Medline, PubMed and Ovid) along with African Journals Online with reported information on cervical cancer and OS and/ or LRR and DFS yielded 410 relevant studies, 45 of which matched the eligibility requirements and were unanimously approved by the two authors (EKD and FIRMIN). We conducted a second searching of possible articles (October 2021) in order to confirm the search results, however no relevant articles were found. Then, a third search was conducted in April 2022, and five additional articles were included.

### Study characteristics

A total of 16,122 women with cervical cancer were covered in the 45 articles, with research sample sizes ranging from 22 to 1,059 (median = 187.5). Western Africa was represented by 12 studies (Ghana = 4, Cote d'Ivoire = 3, Nigeria = 2, Benin = 1, Guinea = 1, Burkina Faso = 1; 2,557 cervical cancer patients:), Southern Africa comprised of 8 studies (South Africa = 5, Botswana = 2, Namibia = 1; 2,482 patients), 13 from Northern Africa (Morocco = 7, Egypt = 4, Tunisia = 2; 1,982 patients), 23 from Eastern Africa (Ethiopia = 5, Uganda = 5, Kenya = 4, Zimbabwe = 4, Malawi = 1, Seychelles = 1, Mozambique = 1, Mauritius = 1, Tanzania = 1; 8,218 patients), 1 from Central Africa (Cameroon; 213 patients). Two studies were multi-regional and the authors were not African hence was considered mixed (670 patients). Seven studies reported DFS and LRR. Table 2 shows the details of the selected studies (27–70).

**Table 2:** Characteristics of the selected studies.

**Author**	**Year**	**Study design**	**Country**	**Sub-regions**	**Race**	**Sample size**
Samaila	2022	Prospective	Nigeria	Western	Black African	157
Aka	2021	Retrospective	Cote d'Ivoire	Western	Black African	39
Scott	2021	Retrospective	Ghana	Western	Black African	420
Sengayi-Muchengeti-1	2020	Retrospective	Benin	Western	Black African	38
Sengayi-Muchengeti-2	2020	Retrospective	Cote d'Ivoire	Western	Black African	200
Vulpe	2018	Retrospective	Ghana	Western	Black African	250
Nartey	2017	Retrospective	Ghana	Western	Black African	821
Camara	2017	Retrospective	Guinea	Western	Black African	311
Toure	2017	Retrospective	Cote d'Ivoire	Western	Black African	78
Toure	2017	Retrospective	Burkina Faso	Western	Black African	78
Musa	2016	Retrospective	Nigeria	Western	Black African	65
Opoku	2016	Retrospective	Ghana	Western	Black African	100
MacDuffie	2021	Prospective	Botswana	Southern	Black African	143
Sengayi-Muchengeti-8	2020	Retrospective	Namibia	Southern	Black African	74
Sengayi-Muchengeti-9	2020	Retrospective	South Africa	Southern	Black African	931
Simonds	2018	Prospective	South Africa	Southern	Black African	492
Grover	2018	Prospective	Botswana	Southern	Black African	143
Ralefala	2018	Retrospective	South Africa	Southern	Black African	373
Jemu	2018	Retrospective	South Africa	Southern	Black African	228
Mangena	2015	Retrospective	South Africa	Southern	Black African	98
Abdelsalam	2021	Retrospective	Egypt	Northern	Non Black African	60
Bouraoui	2021	Retrospective	Tunisia	Northern	Non Black African	41
Séka	2020	Retrospective	Morocco	Northern	Non Black African	133
Elmajjaoui	2016	Retrospective	Morocco	Northern	Non Black African	646
Sahli	2016	Retrospective	Morocco	Northern	Non Black African	293
Elmarjany	2015	Retrospective	Morocco	Northern	Non Black African	162
Salem	2015	Retrospective	Egypt	Northern	Non Black African	83
El-Hadaad	2015	RCT	Egypt	Northern	Non Black African	45
Khalil	2015	Retrospective	Morocco	Northern	Non Black African	303
Errihani	2011	RCT	Morocco	Northern	Non Black African	22
Refaat	2011	Retrospective	Egypt	Northern	Non Black African	40
Chargui	2006	Retrospective	Tunisia	Northern	Non Black African	79
Sahraoui	2002	Retrospective	Morocco	Northern	Non Black African	75
Khamis	2021	Retrospective	Tanania	Eastern	Black African	202
Kavuma	2021	Retrospective	Uganda	Eastern	Black African	414
Chibonda	2021	Retrospective	Zimbabwe	Eastern	Black African	226
Sengayi-Muchengeti-3	2020	Retrospective	Ethiopia	Eastern	Black African	214
Sengayi-Muchengeti-4	2020	Retrospective	Kenya	Eastern	Black African	145
Sengayi-Muchengeti-5	2020	Retrospective	Kenya	Eastern	Black African	939
Sengayi-Muchengeti-6	2020	Retrospective	Mauritius	Eastern	Black African	428
Sengayi-Muchengeti-7	2020	Retrospective	Mozambique	Eastern	Black African	112
Sengayi-Muchengeti-10	2020	Retrospective	Seychellesa	Eastern	Black African	43
Sengayi-Muchengeti-11	2020	Retrospective	Uganda	Eastern	Black African	151
Sengayi-Muchengeti-12	2020	Retrospective	Zimbabwe	Eastern	Black African	58
Sengayi-Muchengeti-13	2020	Retrospective	Zimbabwe	Eastern	Black African	197
Wu	2020	Prospective	Uganda	Eastern	Black African	149
Moelle	2018	Retrospective	Ethiopia	Eastern	Black African	788
Moelle	2018	Retrospective	Ethiopia	Eastern	Black African	788
Wassie	2018	Retrospective	Ethiopia	Eastern	Black African	634
Kantelhardt	2014	Retrospective	Ethiopia	Eastern	Black African	1,059
Msyamboza	2014	Retrospective	Malawi	Eastern	Black African	178
Khaemba	2013	Retrospective	Kenya	Eastern	Black African	211
Maranga	2013	Retrospective	Kenya	Eastern	Black African	209
Gondos	2005	Retrospective	Uganda	Eastern	Black African	149
Chokunonga	2004	Prospective	Zimbabwe	Eastern	Black African	284
Wabinga	2003	Retrospective	Uganda	Eastern	Black African	261
Tebeu	2021	Retrospective	Cameroon	Central	Black African	213
Griesel	2021	Retrospective	Mixed	Mixed	Black African	632
Einstein	2019	RCT	Mixed	Mixed	Black African	38

### Analysis of overall survival

**Figure 2:**
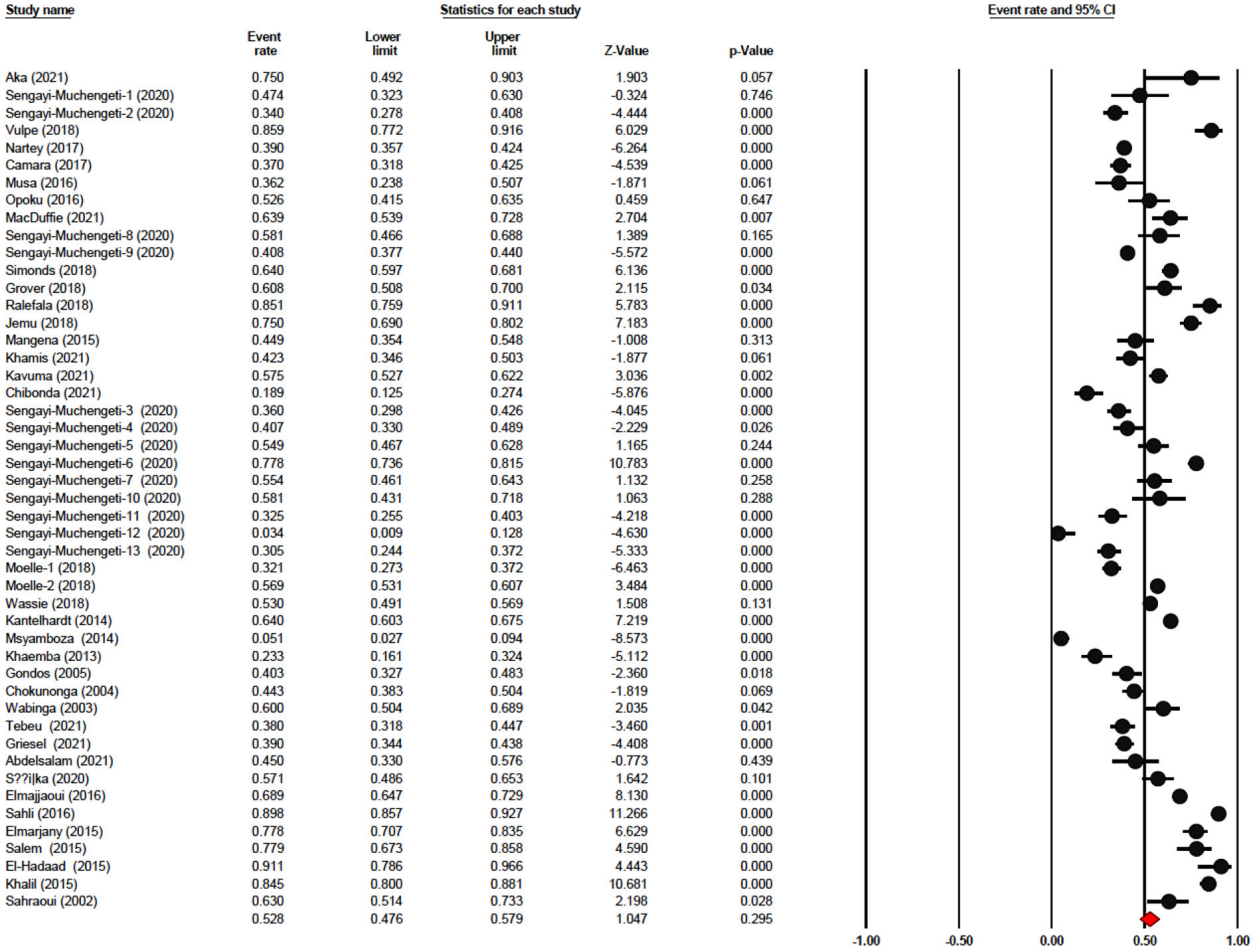
Forest plot showing the 3-year OS.

**Figure 3:**
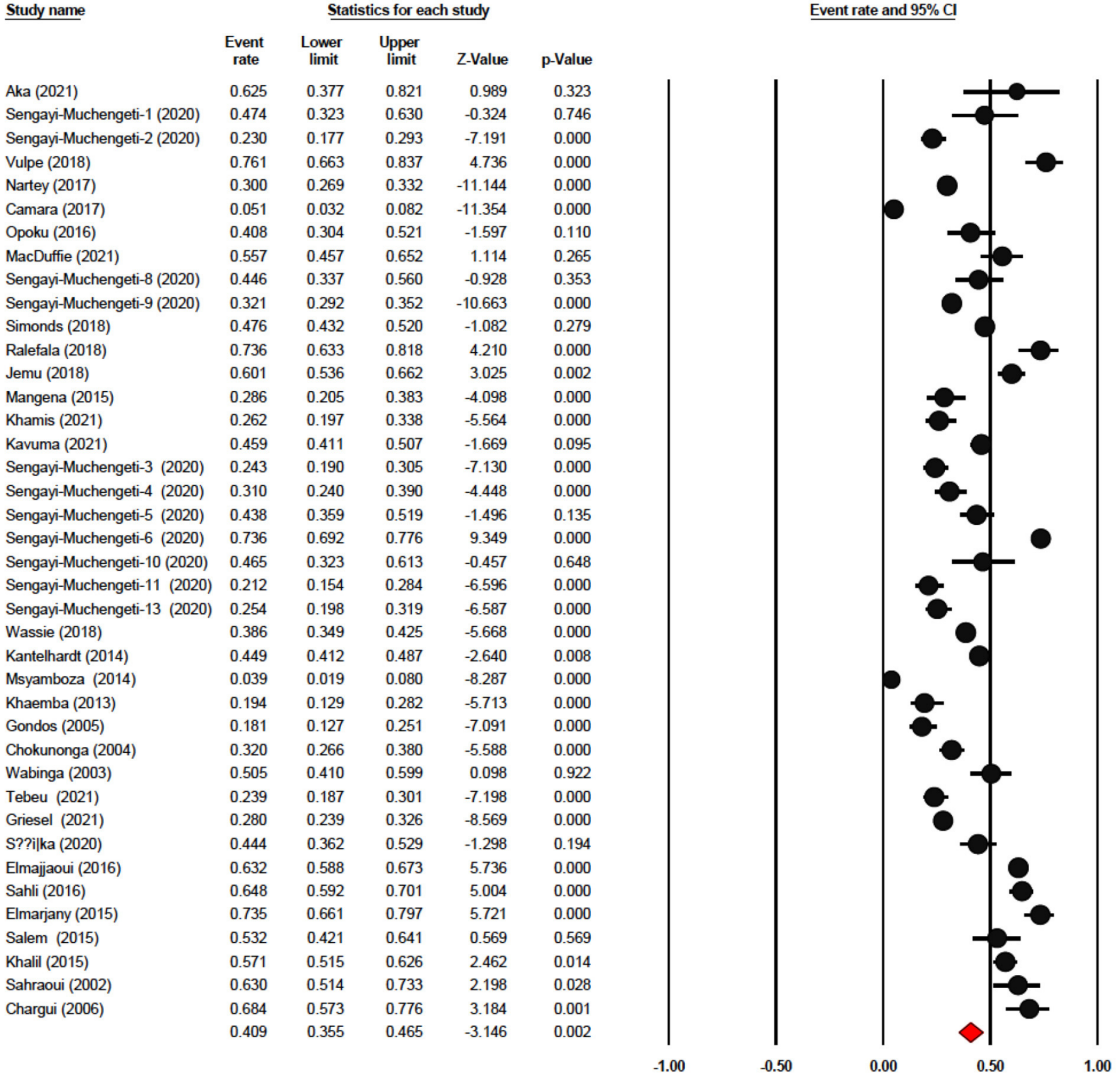
Forest plot showing the 5-year OS.

The one-year survival rate was reported by 45 articles with 16,122 cervical cancer patients as sample size. The one-year survival rate was 77.5% (95% CI: 73.4–81.1%). “Between-study variation in the 1-year survival rates was high (I^2^ = 95.4%; p for heterogeneity <0.001”). The three-year survival rate was reported by 36 articles with 11,208 cervical cancer patients as sample size. The three-year survival rate was 52.8% (95% CI: 47.6–57.9%) (Figure 2). “Between-study variation in the 3-year survival rates was high (I^2^ = 96.0%; p for heterogeneity <0.001”). The five-year survival rate was reported by 30 articles with 9,778 cervical cancer patients as sample size. The five-year survival rate was 40.9% (95% CI: 35.5–46.5%). The five-year survival rate ranged from 3.9% (95% CI: 1.9–8.0%) in Malawi (18) to as high as 76.1% (95% CI: 66.3–83.7%) in Ghana (Figure 3). Between-study variation in the 5-year survival rates was high (I^2^ = 96.2%; p for heterogeneity <0.002).

### Analysis of disease-free survival

**Figure 4:**
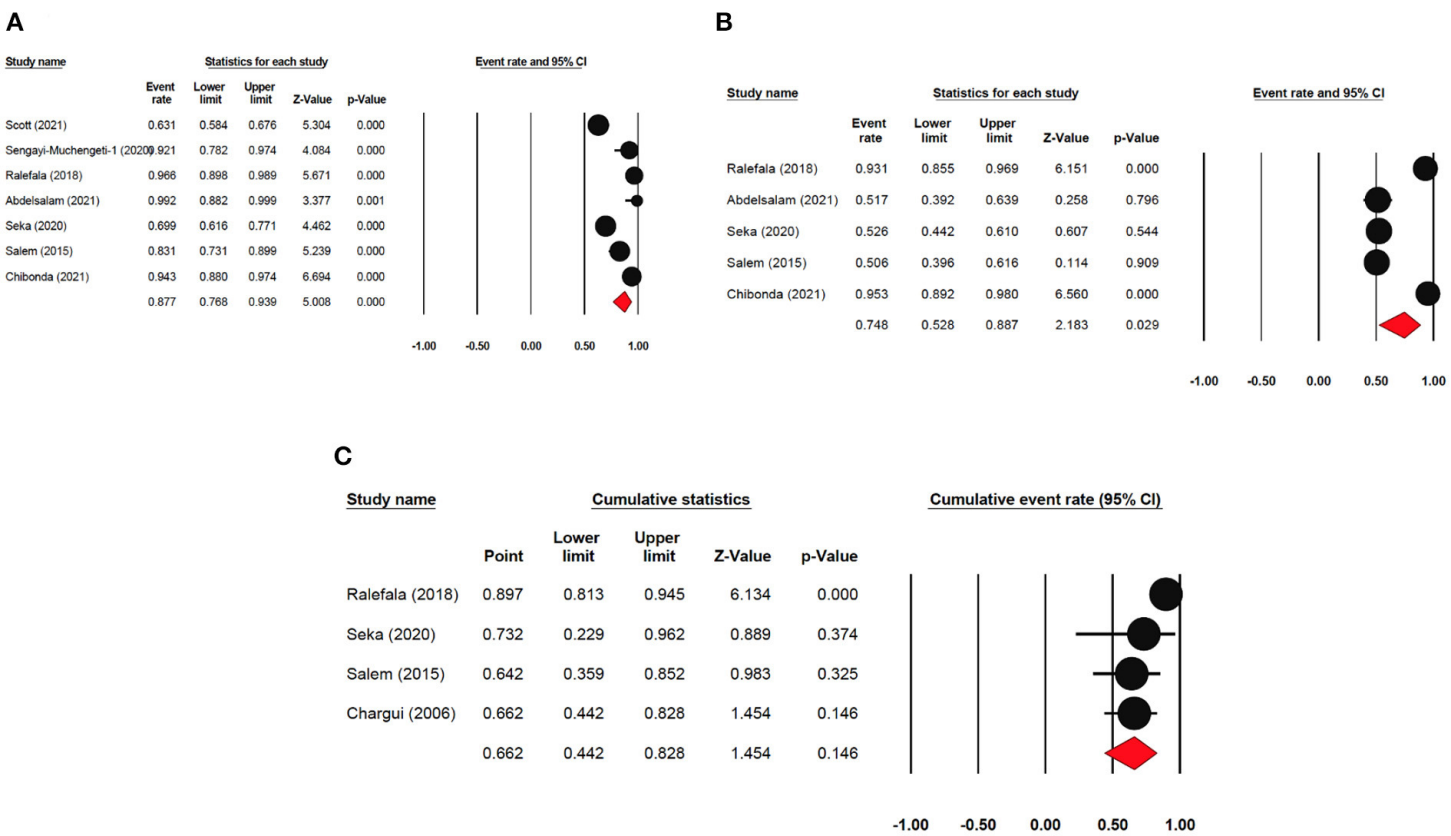
Forest plot showing the DFS. **(A)** pooled 1-year DFS; **(B)** pooled 3-year DFS; **(C)** pooled 5-year DFS.

The one-year disease-free survival rate was reported by 7 articles with 1,333 cervical cancer patients as sample size. The one-year disease-free survival rate was 87.7% (95% CI: 76.8–93.9%) (Figure 4). The three-year disease-free survival rate was reported by 5 articles with 875 cervical cancer patients as sample size. The three-year disease-free survival rate was 74.8% (95% CI: 52.8–88.7%). The five-year disease-free survival rate was reported by 4 articles (4 studies) with 376 cervical cancer patients as sample size. The five-year disease-free survival rate was 66.2% (95% CI: 44.2–82.8%). The five-year disease-free survival rate ranged from 64.2% (95% CI: 35.9–85.2%) to as high as 89.7% (95% CI: 81.3–94.5%).

### Analysis of locoregional rate survival

**Figure 5:**
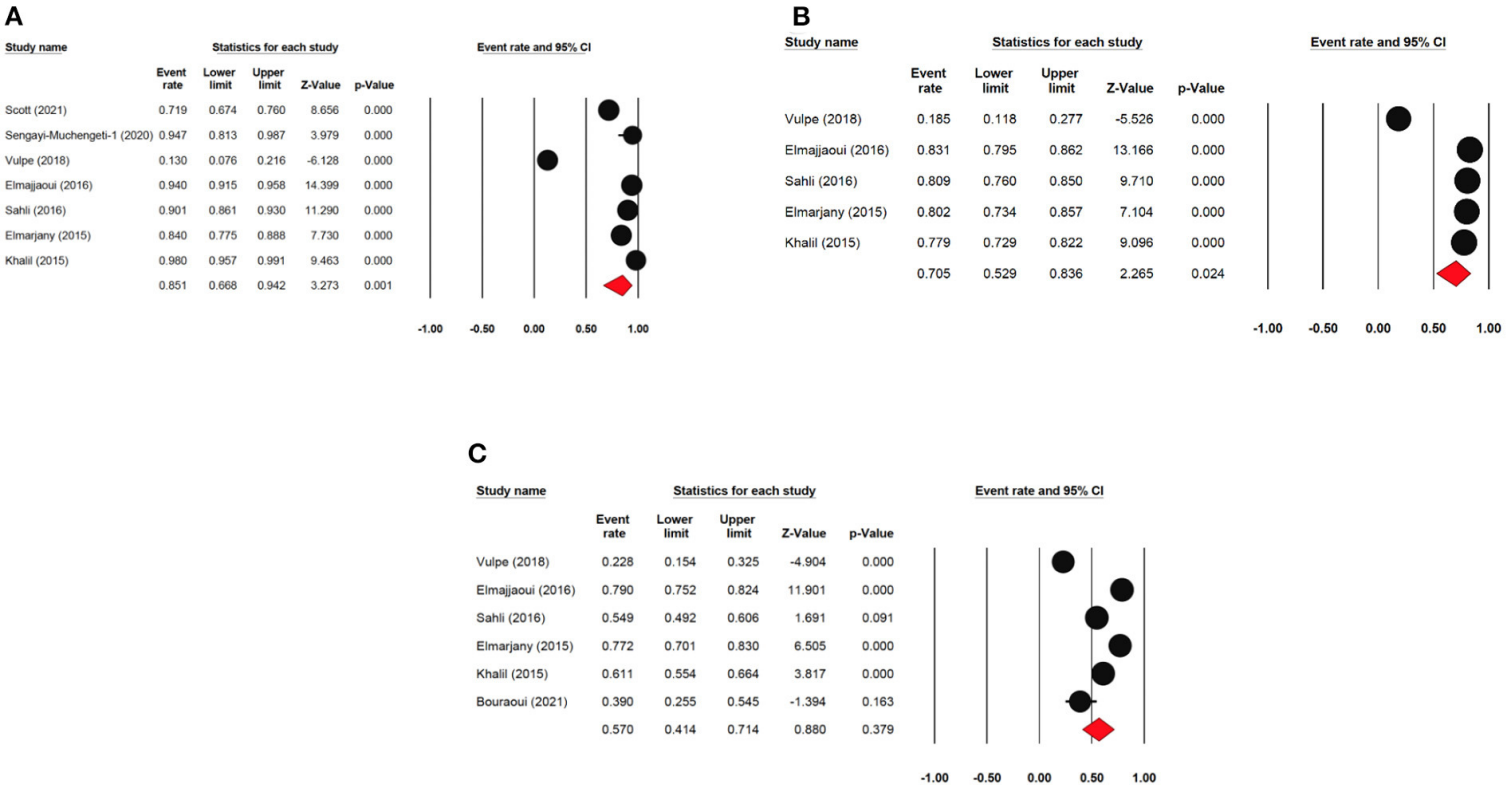
Forest plot showing the LRR. **(A)** pooled 1-year DFS; **(B)** pooled 3-year DFS; **(C)** pooled 5-year DFS.

The one-year locoregional rate survival was reported by seven articles with 1,794 cervical cancer patients as sample size. The one-year locoregional rate survival was 85.1% (95% CI: 66.8–94.2%). The three-year locoregional rate survival was reported by five articles with 1336 cervical cancer patients as sample size. The three-year locoregional rate survival was 70.5% (95% CI: 52.9–83.6%). The five-year locoregional rate survival was reported by 6 articles (6 studies) with 1,377 cervical cancer patients as sample size. The five-year disease-free survival rate was 57.0% (95% CI: 41.4%−88.7%) (Figure 5). The five-year locoregional rate survival ranged from 22.8% (95% CI: 15.4–32.5%) to as high as 79.0% (95% CI: 75.2–82.4%).

### Meta-regression

**Figure 6:**
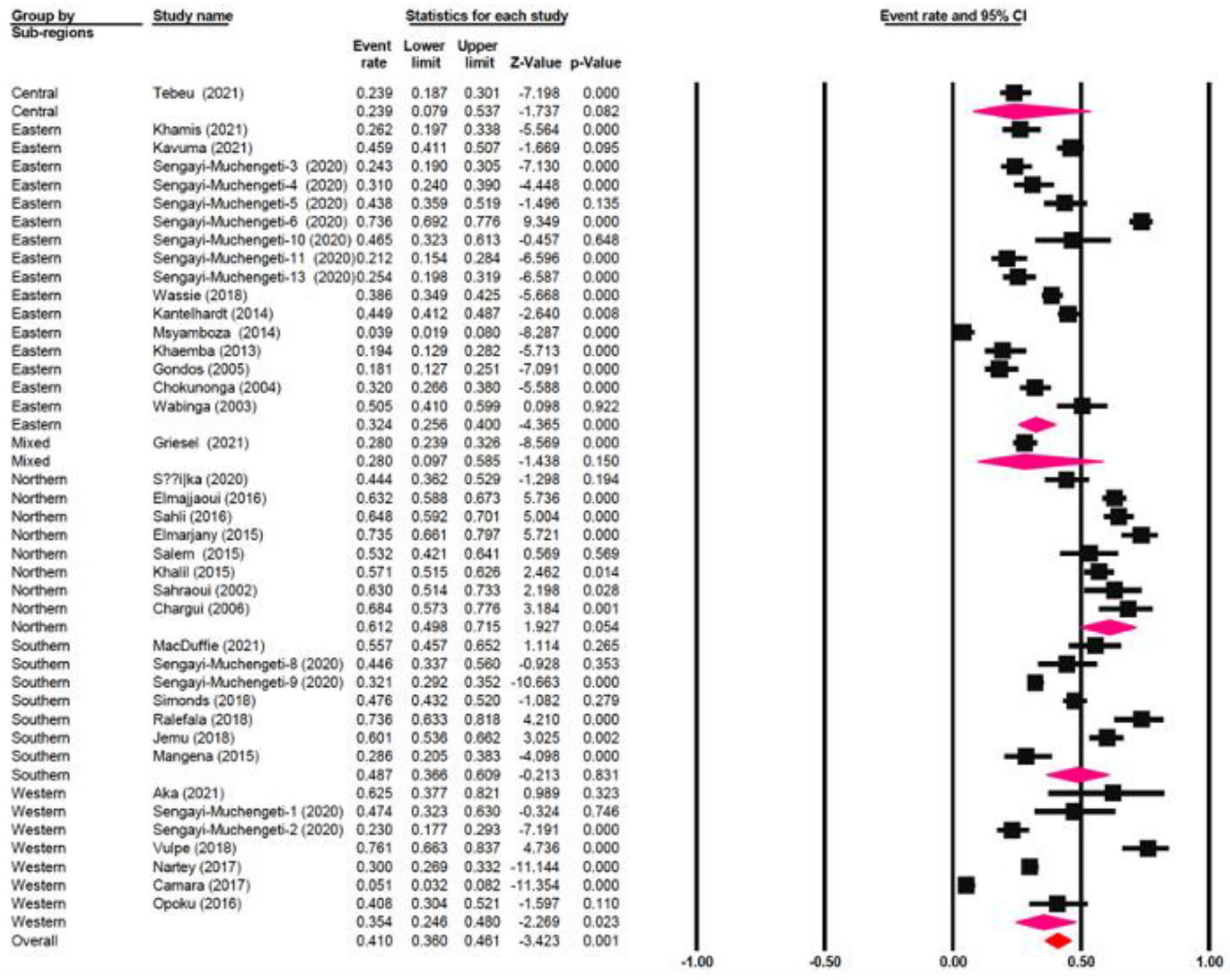
Forest plot of regional sub-groups.

**Figure 7:**
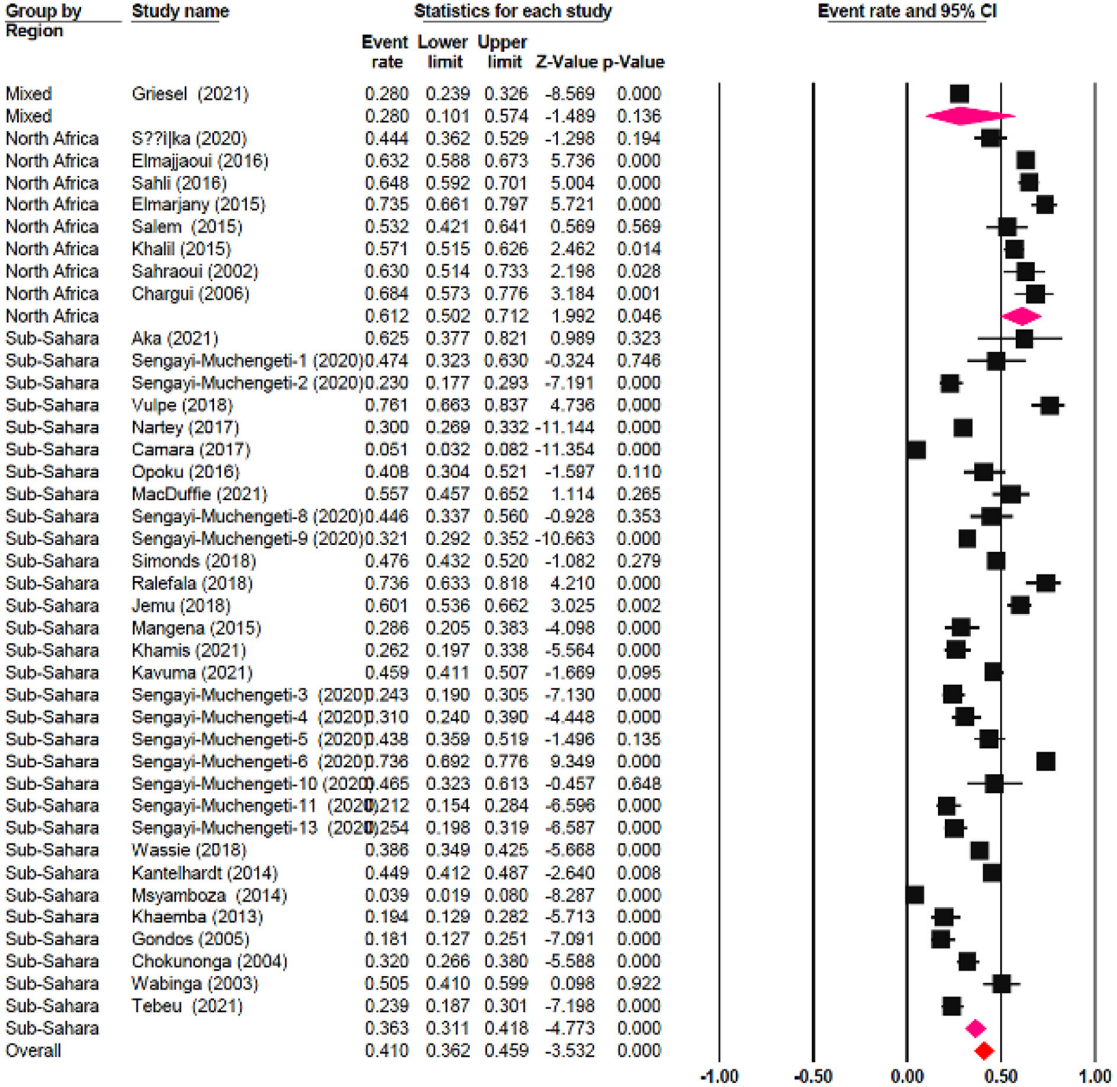
Forest plot on racial sub-groups.

The meta-regression evaluation is represented in Table 3. In comparison to north Africa, survival rates in Sub-Saharan Africa were 24.6% lower (95 % CI: −33.80, −17.28 %). Furthermore, survival rates were statistically significantly different among sub-regions: Southern African had a survival rate of 12.5% lower (95% CI: −22.31, 4.49%), Central African patients had survival rate of 37.3% lower (95% CI: −65.98, 14.39%), Eastern African patients had a survival rate of 28.8% lower (95% CI: −38.24, 9.65%), and Western African patients had a survival rate of 25.8% lower (95 percent CI: −38.48, 13.77%) than Northern African patients (Figure 6). Additionally, survival in the black-African race was 24.9% lower (95% CI: −30.54, −15.34%) (Figure 7), compared to non-black African race.

**Table 3:** Meta-regression analysis of gender, age, and race.

**Survival rate predicators**	**Sample size**	**Adjusted difference in survival rate**	***p*–value**
**Age**	16,122	0.168 (−0.338, 0.584)	0.012
**Sub-region**			
Mixed	410	−1.399 (−2.765, −0.033)	0.045
Southern	2,007	−0.511 (−1.191, 0.170)	0.141
Eastern	3,988	−1.192 (−1.762, −0.622)	< 0.001
Western	1,554	−1.058 (−1.754, −0.361)	0.003
Central	213	−1.613 (−0.008, 0.922)	0.06
Northern	1,606	Reference	
**Region**			
Mixed	410	−1.399 (−2.718, −0.080)	0.038
Sub-Sahara	7,762	−1.020 (−1.525, −0.514)	0.001
Northern Africa	1,606	Reference	
**Race**			
Black African	7,762	−1.019 (−1.524, −0.514)	0.001
Mixed	410	−1.399 (−2.718, −0.080)	0.038
Non-Black African	1,606	Reference	

Furthermore, age had no bearing on the heterogeneity when OS was considered. Squamous carcinoma was correlated with 5-year survival rate (Pearson correlation coefficient r = −0.27, p = 0.02) and stage was correlated with 5-year survival rate (r = 0.241, p = 0.04).

### Heterogeneity sources

The I^2^ metric determines how much of overall variability is influenced by heterogeneity, whereas the Cochran's Q metric determines if the same effect was evaluated by all studies. The I^2^ value of 95.5–96.2% and the heterogeneity chi-square test (*p* < 0.01) both suggested significant heterogeneity among the evaluated studies. The heterogeneity was not explained by sub-group evaluation based on sub-region, country, and region. For subgroup differences, chi-squared statistical analysis consistently gave *p* < 0.05.

### Publication bias

**Figure 8:**
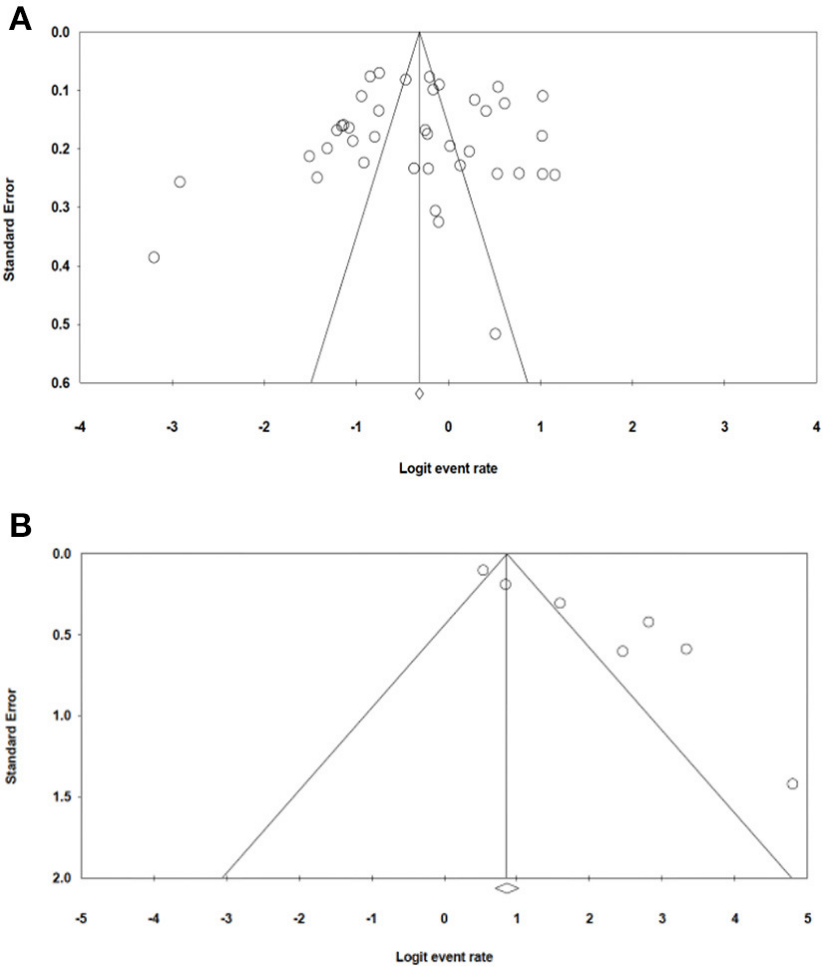
**(A,B)** Funnel plot of publication bias.

The bias in publication bias of the studies selected in our study was evaluated using Egger's test and Begg's funnel plot. The symmetrical funnel plot for each survival rate revealed that publication bias had no effect on the results of our study (Figure 8). Additionally, the results of the Egger's test revealed no evidence of bias existed among our chosen studies, as all of the survival rate *p* > 0.05 [p = 0.756 (OS); *p* = 0.292 (LRR); *p* = 0.138 (DFS)].

### Sensitivity analysis

**Figure 9:**
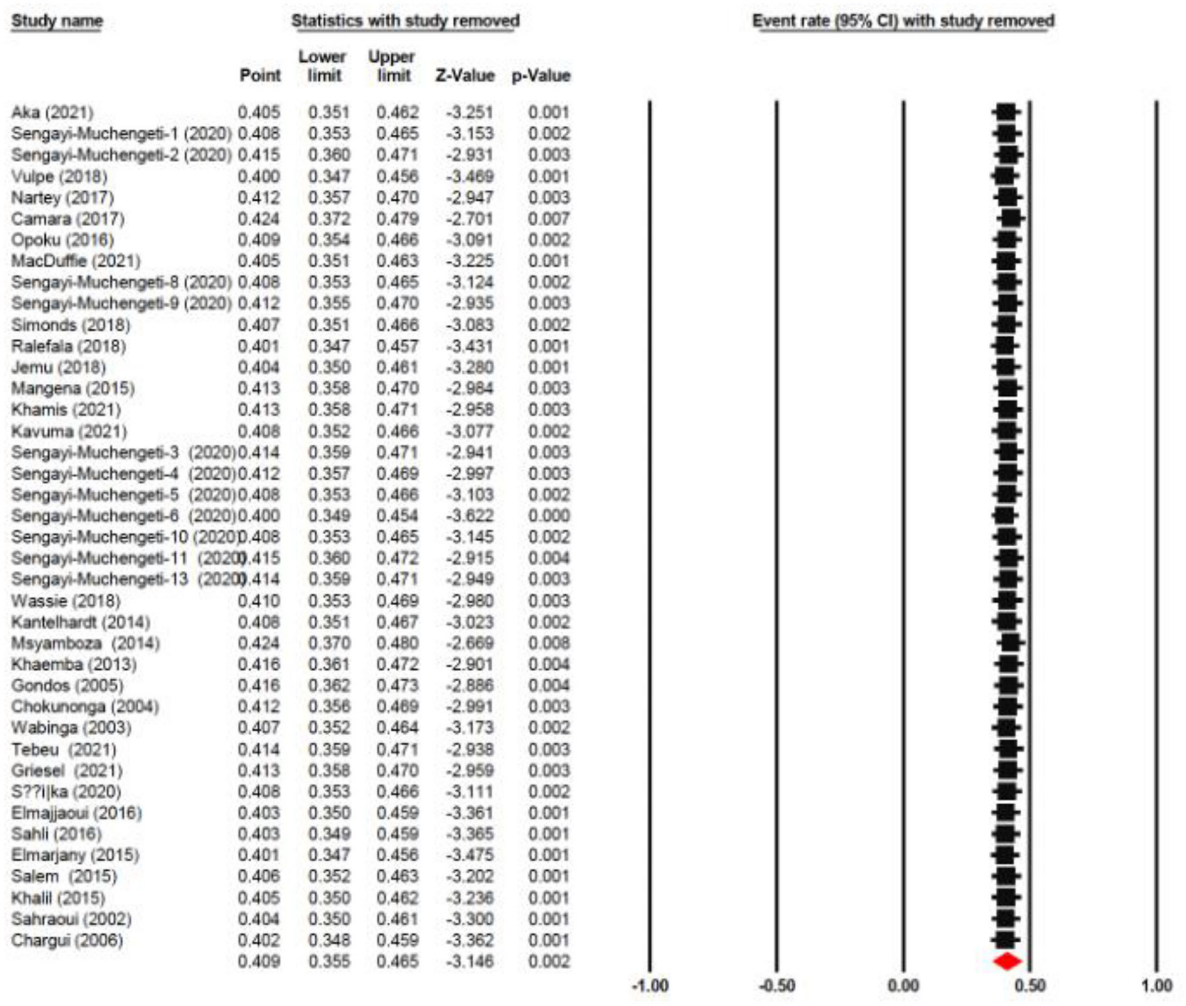
Sensitivity analysis plot of pooled survival.

In order to assess the stability of our findings, we utilized sensitivity analysis (Figure 9). The stability of our study findings was then assessed using a sensitivity analysis. When a particular article was removed from the study, the statistical significance of the findings did not alter, demonstrating the validity and consistency of our findings.

### Newcastle Ottawa Scale assessment of quality of evidence

Where the study designs of the selected articles were retrospective, or prospective to minimize bias, the findings offered high quality evidence of the pooled survival. No statistically significant variations was observed in the pooled estimation based on the studies' level of bias. Eight (8) studies were found in the influence analysis as having a substantial but minor impact on the overall estimation since their sample size was <50.

Nevertheless, due to the significant variability, the evidence is assessed as extremely poor in consistency and high in accuracy owing to the narrow confidence intervals created by the high sample size. We are reasonably optimistic in the applicability or generalizability of several studies since they used representative populations taken from the general populace. We have high confidence in the outcomes in the publication bias because the Egger's test and the funnel plots did not reveal enough indications of selection and reporting bias.

## Discussion

The main purpose of this study was to establish an up-to-date assessment of cervical cancer survival rates in Africa. Our study is the first to solely evaluate the pooled survival rate of cervical cancer patients living in Africa. We calculated the 5-year survival times of cervical cancer across the African region. We combined information from 45 studies that included 16,122 patients diagnosed with cervical cancer with follow-up information to assess chances of survival. Our findings show that focusing at survival time at a regional or global perspective may obscure the distinct disparities in global burden of disease across the sub-regions. The most significant outcome is that the 5-year survival rate, DFS and LRR after being diagnosed with cervical cancer on the African continent is 41%, 66% and 57% respectively.

The 5-year OS in Northern African was 25% higher than all Sub-Saharan African countries. It's important emphasizing that a significant number of black people are residing in the Sub-Sahara countries as contrary to the significant number of non-black people residing in Northern African countries. The central zone of Sub-Saharan Africa had the lowest 5-year OS, at 24%, preceded by 32% from the eastern zone, 35% from the western zone and 49% from the southern zone. This could be partly attributed to the differences in government healthcare expenditure and socioeconomic status across various zones. As proven by a significantly higher governmental healthcare expenditure (% of GDP) of 11.27 in Lesotho in 2019 comparison to Ghana's 3.42%, Kenya's 4.59%, Egypt's 4.74%, Cameroon 3.60% and Angola 2.53% (71). The southern zone has a higher governmental healthcare expenditure and a thriving economy then either western, eastern or central Africa (72). The state of a country's economy is linked to certain variables that are established to affect survival, such as nutrition, psychosocial wellbeing, accessibility to healthcare, and the stage at which a person is diagnosed. Ingelby et al. reported that an association existed between cancer survival and socioeconomic status (73). Cervical cancer survival rate on the continent of Africa is lower than other developed nations. Lower levels of cervical cancer knowledge, as well as other obstacles to health services accessibility, such like longer distances between health-care institutions, are important determinants of late diagnosis and associated poor survival rates in Sub-Saharan Africa (74–77). The lower survival rate observed is due to gaps management capacities, diagnostic, screening, prevention and late diagnosis. Nearly half of all people on the planet lack access to diagnostics (78). Diagnostics are essential to providing high-quality medical care. This idea is not widely accepted, which results in underinvestment and insufficient resources at all stages. Primary healthcare is the so-called “final mile” of diagnostic care and notably affects marginalized, rural and poor communities worldwide; equitable accessibility is crucial for social justice (78, 79). Without effective and proper diagnostics accessibility, it is impossible to provide comprehensive healthcare services and be prepared for pandemics, as has been highlighted by the COVID-19 pandemic. The diagnostic disparity can be decreased, access improved, and diagnostics can be made more accessible for patients by considering a variety of fields (such as workforce, technology and financing). By decreasing the diagnostic gap, one million untimely deaths in developing nations could be prevented each year. Randall et al. reported that there are significant differences in both the technologies accessible and the degree of accessibility to cervical cancer screening among the healthcare institutions in Sub-Saharan Africa (80).

Elmore et al. reported that four hundred thirty (430) megavoltage units were available in Africa as of March 2020, and the operational capacity increased by 45% compared to 2012 (81). Of the 54 African nations, 28 (52%) had access to “external beam radiotherapy (EBRT)”, whereas, in 2012, EBRT was accessible in 23 African nations (43%). Two nations, South Africa (97 units) and Egypt (119 units), accounted for approximately 50% of the units installed. In Mauritius, there were 2.37 “megavoltage units per million people”, compared to 0.02 in Uganda, Nigeria, and Ethiopia. For nations offering radiation therapy treatments, the megavoltage units per 1,000 cancer patients ranged from 1.38 in Tunisia to 0.01 in Ethiopia. Tunisia, Mauritius, and Gabon were the only three nations with at least one megavoltage unit per 1,000 cancer cases (81).

Of the 430 megavoltage units in 2020, linear accelerators (LINACs) made almost 85% of those units. Furthermore, the quantity of linear accelerators surged by 78% since 2012, and the quantity of cobalt-60 teletherapy units declined by 28% (81). Presently, there are about 5.7 LINAC per cobalt-60 teletherapy unit. LINACs and Cobalt-60 units are both present in about half of all nations. LINACs only are used in 11 (20%) nations, while cobalt-60 units are used in 3 (11%) nations (81). Megavoltage units are now more widely available in all locations. The improvement in coverage was limited (apart from northern Africa) and even reduced in southern Africa amid this capacity increase.

Of the 54 African countries, only 21 (39 %) have the necessary resources to provide brachytherapy (BT) treatments. About 102 installed brachytherapy units were the resources that involved both low-dose-rate and high-dose-rate brachytherapy units. The countries with the most installed brachytherapy units South Africa (24), Egypt (23), and were Algeria (12), which collectively accounted for about 60% of the total brachytherapy (BT) units. Of the overall brachytherapy equipment in Africa, high-dose-rate BT makes up 68 % (70 afterloaders). Considering that cervical cancer frequently presents in the advanced stages, the existing BT units can be used in treating about 36,100 women diagnosed with cervical cancer annually. Furthermore, the utilization rate of brachytherapy if all units available were devoted to treating patients was about 75%. Thus, the operational output of brachytherapy meets 37% of the continent's healthcare needs (81).

There is now a massive shortage of megavoltage units, with ~1,018 megavoltage units short of what would be required to service over 1 million cancer patients. According to the region, 53 units are needed in southern Africa, central Africa 122, northern Africa 123, western Africa 308, and 412 in Eastern Africa to meet the existing demand. Nigeria has the most significant demand in west Africa (147 more megavoltage units are required), accounting for 14% of the total demand in Africa and 47% of the demand in West Africa (81).

Cervical cancer mortality can be reduced by implementing cost-effective screening programs in low-resource areas. The results of the 12-month feasibility study, which took place between September 2013 and October 2014, show that cervical cancer screening programs employing HPV testing was successfully launched and sustained in resource-constrained areas in Rwanda. The program was effectively implemented amidst initial difficulties, such as logistic and financial issues, follow-up loss, patient reluctance and care providers training.Developing nations must integrate cervical cancer treatment, screening and HPV vaccination into regular and routine healthcare services for women. Thorough evaluation and monitoring, creative collaborations, cross-sectoral planning and coordination and political will are all necessary for this (82–84). Any cervical cancer preventive initiative must tackle both system and patients-centered challenges. Comprehensive cervical cancer management in Sub—Saharan Africa is hampered by inadequate infrastructure and limited human capacity (79). Multi-disciplinary team comprising Gynecology oncology, medical oncology, radiology, pathology, radiation oncology, and palliative care are all involved in the management of metastatic, locally advanced or early-stage cervical cancer. The treatment of cervical cancer is multimodal; early-stage can be treated with curative oncologic surgery while recurrent or advanced cancer requires chemotherapy or radiation treatment. The current capacity of SSA nations to offer comprehensive women's cancer treatment is limited due to a scarcity of experienced and trained in surgical oncologist. In SSA, the difficulty of health-care human resources affects all sectors of the healthcare workforce, and new strategies in increasing the capability and capacity of the healthcare staff centered on each nation's unique circumstances are critical to any nationwide cancer control effort. The causes for the significant survival disparities between populations North Africans and in Sub-Saharan Africans, as well as between non-blacks and blacks observed in our study are not totally apparent. Despite the fact that the majority of patients were 20–45 years old at the time of diagnosis, we found a significant correlation between overall survival and age at diagnosis (85, 86).

The large sampling size of about 16,000 women with cervical cancer in Africa is one of the study's main novelties. There were, however, certain setbacks. Firstly, we were only able to incorporate publications from 23 African nations out of the total 54 African nations. This can be attributed to lack of available information. Secondly, the accessible studies only covered individuals who reported at health-care institutions, primarily tertiary facilities. Across the African continent, accessibility to healthcare facilities continues to be a major factor in poor treatment adherence and delayed diagnosis.

The 5-year overall survival rates of cervical cancer in African black race were lower than in both white and black race in the United States. As a result, one of the cervical cancer control preventive measures employed on this continent ought to be early detection of cervical cancer *via* cervical cancer education activities such as screening and HPV vaccination along with enhanced management. Eradicating barriers associated with the diagnosis of cervical cancer and strategies resulting in earlier diagnosis, coupling with adequate management and timely intervention, can increase cervical cancer survival on African continent in the future.

## Conclusion

In conclusion, we found racial, and sub-regional variations in cervical cancer survival rates over the whole African continent. To enhance cervical cancer survival, geographical and racial group health promotion measures, as well as prospective genetic investigations, are critically required.

## Data availability statement

The raw data supporting the conclusions of this article will be made available by the authors, without undue reservation.

## Author contributions

ED wrote and presented the original draft. ED and FF were involved in data curation and visualization. CE, CA, YZ, FA, and M-AD were involved in methodology, software, analysis, review, and editing. WX, YW, and KS were involved in supervision. All authors contributed to the article and approved the submitted version.

## Funding

This study was partially supported by the National Natural Science Foundation of China (Nos. 81971508, 81471589, and 81273259), the Health Bureau of Henan Province, China (No. 201201005), and the Foundation and Frontier Research Grant of Henan Provincial Science and Technology Bureau, China (Nos. 112300410027 and 132102310120).

## Conflict of interest

The authors declare that the research was conducted in the absence of any commercial or financial relationships that could be construed as a potential conflict of interest. The reviewer JR declared a past co-authorship/collaboration (https://www.frontiersin.org/articles/10.3389/fimmu.2022.853352/full) with the author ED to the handling editor.

## Publisher's note

All claims expressed in this article are solely those of the authors and do not necessarily represent those of their affiliated organizations, or those of the publisher, the editors and the reviewers. Any product that may be evaluated in this article, or claim that may be made by its manufacturer, is not guaranteed or endorsed by the publisher.
