# Supplementary material for: Cervical cancer survival times in Africa
Source: Front Public Health. 2022 Nov 9;10:981383. doi: 10.3389/fpubh.2022.981383 (PMC9683338; doi:10.3389/fpubh.2022.981383)
Supplement: Supplementary file 1 [file Table_1.docx]

# Table S1. Detailed Newcastle-Ottawa Scale of each included cohort study.

|  | Selection | | | | Comparability | | Outcome | | |  |
| --- | --- | --- | --- | --- | --- | --- | --- | --- | --- | --- |
| Study | Representativ eness of exposed cohort | Selection of non- exposed cohort | Ascertainment of exposure | Demonstration that outcome  of interest was not present at start of  study | Adjust for the most important risk factors | Adjust for other risk factors | Assessment of outcome | Follow-up length | Loss to follow-up rate | **Total quality score** |
| Samaila 2022 | 1 | 0 | 1 | 1 | 0 | 1 | 1 | 1 | 1 | **7** |
| Aka 2021 | 1 | 0 | 1 | 1 | 0 | 1 | 1 | 1 | 0 | **6** |
| Scott 2021 | 1 | 1 | 1 | 1 | 1 | 1 | 1 | 1 | 1 | **9** |
| Sengayi-Muchengeti 2020 | 1 | 0 | 1 | 1 | 0 | 1 | 1 | 1 | 1 | **7** |
| Vulpe 2018 | 1 | 0 | 1 | 1 | 0 | 1 | 1 | 1 | 0 | **6** |
| Nartey 2017 | 1 | 1 | 1 | 1 | 1 | 1 | 1 | 1 | 1 | **9** |
| Camara 2017 | 1 | 0 | 1 | 1 | 0 | 1 | 1 | 1 | 1 | **7** |
| Toure 2017 | 1 | 0 | 1 | 1 | 0 | 1 | 1 | 1 | 1 | **7** |
| Musa 2016 | 1 | 0 | 1 | 1 | 0 | 1 | 1 | 1 | 0 | **6** |
| Opoku 2016 | 1 | 0 | 1 | 1 | 0 | 1 | 1 | 1 | 0 | **6** |
| MacDuffie 2021 | 1 | 1 | 1 | 1 | 1 | 1 | 1 | 1 | 0 | **8** |
| Simonds 2018 | 1 | 1 | 1 | 1 | 1 | 1 | 1 | 1 | 1 | **9** |
| Grover 2018 | 1 | 1 | 1 | 1 | 1 | 1 | 1 | 1 | 0 | **8** |
| Ralefala 2018 | 1 | 1 | 1 | 1 | 1 | 1 | 1 | 1 | 0 | **8** |
| Jemu 2018 | 1 | 0 | 1 | 1 | 0 | 1 | 1 | 1 | 1 | **7** |
| Mangena 2015 | 1 | 1 | 1 | 1 | 1 | 1 | 1 | 1 | 1 | **9** |
| Abdelsalam 2021 | 1 | 0 | 1 | 1 | 0 | 1 | 1 | 1 | 1 | **7** |
| Bouraoui 2021 | 1 | 0 | 1 | 1 | 0 | 1 | 1 | 1 | 1 | **7** |
| Séka 2020 | 1 | 0 | 1 | 1 | 0 | 1 | 1 | 1 | 1 | **7** |
| Elmajjaoui 2016 | 1 | 0 | 1 | 1 | 0 | 1 | 1 | 1 | 0 | **6** |
| Sahli 2016 | 1 | 1 | 1 | 1 | 1 | 1 | 1 | 1 | 1 | **9** |
| Elmarjany 2015 | 1 | 0 | 1 | 1 | 0 | 1 | 1 | 1 | 1 | **7** |
| Salem 2015 | 1 | 0 | 1 | 1 | 0 | 1 | 1 | 1 | 1 | **7** |
| El-Hadaad 2015 | 1 | 0 | 1 | 1 | 0 | 1 | 1 | 1 | 1 | **7** |
| Khalil 2015 | 1 | 0 | 1 | 1 | 0 | 1 | 1 | 1 | 1 | **7** |
| Errihani 2011 | 1 | 0 | 1 | 1 | 0 | 1 | 1 | 1 | 1 | **7** |
| Refaat 2011 | 1 | 0 | 1 | 1 | 0 | 1 | 1 | 1 | 1 | **7** |
| Chargui 2006 | 1 | 0 | 1 | 1 | 0 | 1 | 1 | 1 | 1 | **7** |
| DeBoer 2022 | 1 | 1 | 1 | 1 | 1 | 1 | 1 | 1 | 0 | **8** |
| Khamis 2021 | 1 | 0 | 1 | 1 | 0 | 1 | 1 | 1 | 0 | **6** |
| Kavuma 2021 | 1 | 1 | 1 | 1 | 1 | 1 | 1 | 1 | 1 | **9** |
| Chibonda 2021 | 1 | 0 | 1 | 1 | 0 | 1 | 1 | 1 | 0 | **6** |
| Wu 2020 | 1 | 1 | 1 | 1 | 1 | 1 | 1 | 1 | 1 | **9** |
| Moelle 2018 | 1 | 1 | 1 | 1 | 1 | 1 | 1 | 1 | 0 | **8** |
| Wassie 2018 | 1 | 0 | 1 | 1 | 0 | 1 | 1 | 1 | 1 | **7** |
| Kantelhardt 2014 | 1 | 0 | 1 | 1 | 0 | 1 | 1 | 1 | 0 | **7** |
| Msyamboza 2014 | 1 | 1 | 1 | 1 | 1 | 1 | 1 | 1 | 1 | **9** |
| Khaemba 2013 | 1 | 0 | 1 | 1 | 0 | 1 | 1 | 1 | 0 | **6** |
| Maranga 2013 | 1 | 1 | 1 | 1 | 1 | 1 | 1 | 1 | 1 | **9** |
| Gondos 2005 | 1 | 1 | 1 | 1 | 1 | 1 | 1 | 1 | 1 | **9** |
| Chokunonga 2004 | 1 | 0 | 1 | 1 | 0 | 1 | 1 | 1 | 1 | **7** |
| Wabinga 2003 | 1 | 1 | 1 | 1 | 1 | 1 | 1 | 1 | 0 | **8** |
| Tebeu 2021 | 1 | 1 | 1 | 1 | 1 | 1 | 1 | 1 | 1 | **9** |
| Griesel 2021 | 1 | 0 | 1 | 1 | 0 | 1 | 1 | 1 | 0 | **6** |
| Einstein 2021 | 1 | 0 | 1 | 1 | 0 |  | 1 | 1 | 0 | **6** |

**Good quality:** 3 or 4 stars in selection domain AND 1 or 2 stars in comparability domain AND 2 or 3 stars in outcome/exposure domain

**Fair quality:** 2 stars in selection domain AND 1 or 2 stars in comparability domain AND 2 or 3 stars in outcome/exposure domain

**Poor quality:** 0 or 1 star in selection domain OR 0 stars in comparability domain OR 0 or 1 stars in outcome/exposure domain
