# Supplementary material for: Cervical cancer survival times in Africa
Source: Front Public Health. 2022 Nov 9;10:981383. doi: 10.3389/fpubh.2022.981383 (PMC9683338; doi:10.3389/fpubh.2022.981383)
Supplement: Supplementary file 2 [file Table_2.docx]

**Appendix 1: Search strategy**

| Indicator | Search |
| --- | --- |
| 1 | africa/ or africa, northern/ or algeria/ or egypt/ or libya/ or morocco/ or africa, central/ or cameroon/ or central african/ republic/ or chad/ or congo/ or “democratic republic of the congo”/ or equatorial guinea/ or gabon/ or africa, eastern/ or burundi/ or djibouti/ or eritrea/ or ethiopia/ or kenya/ or rwanda/ or somalia/ or sudan/ or tanzania/ or uganda/ or africa, southern/ or angola/ or botswana/ or lesotho/ or malawi/ or mozambique/ or namibia/ or south africa/ or swaziland/ or zambia/ or zimbabwe/ or africa, western/ or benin/ or burkina faso/ or cape verde/ or cote d'ivoire/ or gambia/ or ghana/ or guinea/ or guinea-bissau/ or liberia/ or mali/ or mauritania/ or niger/ or nigeria/ or senegal/ or sierra leone/ or togo/ African country” or “African countries” or “African nation” or “ African nations” or “Africa country” or “Africa countries” “West African nation” or “West African countries” or “East Africa” or “Eastern African” or “Central Africa” or “North Africa country” or “North Africa countries” or “East Africa nations” or “West Africa countries” |
| 2 | “cervical cancer” or “cervix cancer” or “cancer of the cervix” or “cervical carcinoma” or “cervix tumor” or “cervix tumour” mp. [mp=ti, ab, hw, tn, ot, dm, mf, dv, kw, fx, dq, nm, kf, ox, px, rx, an, ui, sy] |
| 3 | Exp survival/ or overall survival/ or survival rate/ or overall survival rate/ or/ OS (survival* or overall survival* or OS* or survival rate* or overall survival rate* or ).tw,kf |
| 4 | 3 years, 5years, |
| 5 | (comparative or retrospective or prospective or study).mp. [mp=ti, ab, hw, tn, ot, dm, mf, dv, kw, fx, dq, nm, kf, ox, px, rx, an, ui, sy] |
| 6 | Brachytherapy or concurrent or chemoradiotherapy or radiotherapy or radiation therapy or surgery).mp. [mp=ti, ab, hw, tn, ot, dm, mf, dv, kw, fx, dq, nm, kf, ox, px, rx, an, ui, sy] |
| 7 | 1 AND 2 AND 3 |
| 8 | 1 AND 2 AND 4 |
| 9 | 1 AND 2 AND 3 AND 4 |
| 10 | 1 AND 2 |
| 11 | 2 AND 3 AND 6 |
| 12 | 1 AND 2 AND 5 |
| 13 | 1 AND 2 AND 6 |
| 14 | 2 AND 3 AND 5 |
| 15 | Limit 20 to yr “2000-current” |
|  |  |
|  |  |
|  |  |
|  |  |
